# Food Choice and Waste in University Dining Commons—A Menus of Change University Research Collaborative Study

**DOI:** 10.3390/foods10030577

**Published:** 2021-03-10

**Authors:** Tiffany Wiriyaphanich, Jean-Xavier Guinard, Edward Spang, Ghislaine Amsler Challamel, Robert T. Valgenti, Danielle Sinclair, Samantha Lubow, Eleanor Putnam-Farr

**Affiliations:** 1 Department of Food Science and Technology, University of California, Davis, CA 95616, USA; twiriyaphanich@ucdavis.edu (T.W.); esspang@ucdavis.edu (E.S.); 2 Menus of Change University Research Collaborative, Stanford, CA 94305, USA; gchallamel@stanford.edu; 3 Department of Religion and Philosophy, Lebanon Valley College, Annville, PA 17003, USA; valgenti@lvc.edu; 4 Housing Dining & Auxiliary Enterprises—Campus Dining, University of California, Santa Barbara, CA 93106, USA; dsinclair@ucsb.edu; 5 Cal Dining, University of California, Berkeley, CA 94720, USA; smlubow@berkeley.edu; 6 Jones Graduate School of Business, Rice University, Houston, TX 77005, USA; Eleanor.Putnam-Farr@rice.edu

**Keywords:** food choice, food waste, university dining commons, multiple correspondence analysis

## Abstract

The purpose of this multi-campus research was to investigate the relationships of food type and personal factors with food choice, consumption, and waste behaviors of college students at all-you-care-to-eat dining facilities. The amount of food taken and wasted was indirectly measured in units relative to the plate size using before and after photos taken by the diners themselves. Animal protein and mixed dishes (e.g., stir fry, sandwich) took up more of diners’ plate space and these items were correlated to both greater hedonic appeal as well as a higher likelihood of the item being pre-plated. Greater confidence in liking an item before choosing it was correlated to a larger portion being taken. Finally, increased satisfaction with the meal and frequency of visiting the dining commons was correlated to less food waste. Understanding these potential food choice drivers can help dining facilities better target healthier meals to diners while reducing food waste.

## 1. Introduction

Food waste, as well as the prevalence of overweight and obese individuals, has been increasing in the United States [1,2]. Food waste is problematic, given that the negative environmental impacts of investing significant resources in cultivating and processing food items are exacerbated when those items are never eaten. Meanwhile, food waste prevention has been identified as the highest value food waste solution relative to recovery, recycling, and disposal [3]. Consumer food choice and food waste behaviors are complex but must be understood to inform preventative solutions [4,5]. Studies have been conducted to investigate and understand what drives and inhibits healthier eating [6,7], while other studies have been conducted to understand the motivations behind consumer food waste in order to encourage people to waste less food [8,9,10].

University dining commons are great settings to investigate the drivers of food choice and waste because students, still in an evolving stage when it comes to eating, are beginning to form their longer-term food habits, including their potential to engage in food waste reduction [11,12,13]. Many universities have buffet-style/all-you-care-to-eat facilities (AYCE) where diners can take as much food as they desire for a fixed price. This has the advantage of allowing schools to offer more food options and faster service but can result in higher food waste [14]. Interventions using informational prompts [15,16,17], appealing to diners’ social responsibility through cooperation [18,19], encouraging sharing [20], taking away trays [21], changing plate shape [22], and reducing portion amounts [23] have been implemented with some success in reducing food waste. Further, it is important to note that not all food waste carries the same economic and environmental impact. Animal product waste is generally the best target for reduction since it usually costs more and requires the most resources to produce [24].

Reducing food waste needs to be balanced with encouraging university diners to eat healthier foods, specifically vegetables, which provide a protective factor for obesity [25]. Personal factors like cognitive restraint, susceptibility to stress, and gender, as well as environmental factors like time constraints and dining commons design, have been linked to differing food choice behavior [26,27,28]. Nudging interventions, such as changing the presentation of healthy items or adjusting the serving style in university dining commons, have had varying levels of success in getting diners to eat more nutritious foods [29,30,31].

Most of the existing studies that explore food choice and food waste behaviors at universities focus only on a single school. Given the complexity of these issues, studies that utilize multi-campus data have the potential for greater analytical robustness given the increased sample size and captured variance. An intervention’s effects and potential reproducibility can also be seen across a greater population [32]. Though useful, these studies can be logistically challenging in collecting and comparing sample data. One technique used in previous studies to streamline data collection involves taking photos of food as a proxy for other measures of the amount of food taken and wasted [33,34]. This method was utilized in this multi-campus study to identify amounts of quantities of food taken and wasted. These values were then used to investigate the correlations among the food type, the reasons for choosing foods and portions, and the personal factors with the amount of food taken and wasted at university dining commons.

## 2. Materials and Methods

### 2.1. Study Design and Protocol

Five colleges and universities from the Menus of Change University Research Collaborative (MCURC) participated in this study in the Spring of 2019 or the Fall of 2019. The participating schools included Stanford University, the University of California, Berkeley, the University of California, Davis, the University of California, Santa Barbara, and Lebanon Valley College. One school (B) participated in both the Spring and Fall cohorts. Characteristics of these schools are shown in Table 1. Each school had an AYCE dining hall that could be accessed with a card swipe as part of a meal plan, though the different schools demonstrated variation in the average percentage of pre-portioned dishes.

An online survey was designed in Qualtrics and used at all the schools. Rice University’s Institutional Review Board granted exempt status for the study protocol for research activities at all of the campuses. Diners were intercepted at their dining halls and asked if they wanted to participate in a study about food choices and satisfaction, but the objective of investigating food waste behavior was not disclosed. Each participating school determined what incentives would best encourage participation (for example, raffles for gift cards or spinning a wheel for a small prize) and how best to approach students. Both QR codes and tiny urls were used to distribute the survey link. While all studies took place over the same general time periods (Spring or Fall semesters of 2019), the number of data collection days varied among schools depending on how long it took to reach the target number of participants. The goal derived from previous MCURC studies was to collect data from at least 50 people per school which was met at most schools. However, after removing incomplete responses, two schools were unable to reach this quota.

**Figure 1:**
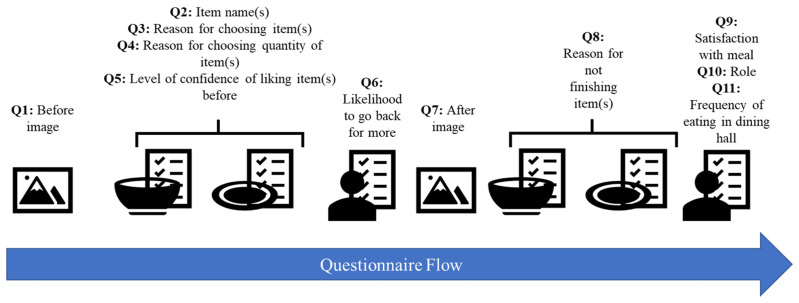
Questions and question type—photo, per dish, per individual—that were asked in this survey.

The survey’s general sequence involved the subject taking a photo of their plate before and after eating, answering a few questions about each item they took (up to six), and responding to some additional questions about themselves. The flow of questions is shown in Figure 1 and specific questions are provided in Table S1. The survey was started after participants had already selected their items so as not to influence their food selection. Similarly, the subjects were not asked why they did not finish their food until after they finished to avoid influencing how much they consumed.

### 2.2. Data Organization

**Figure 2:**
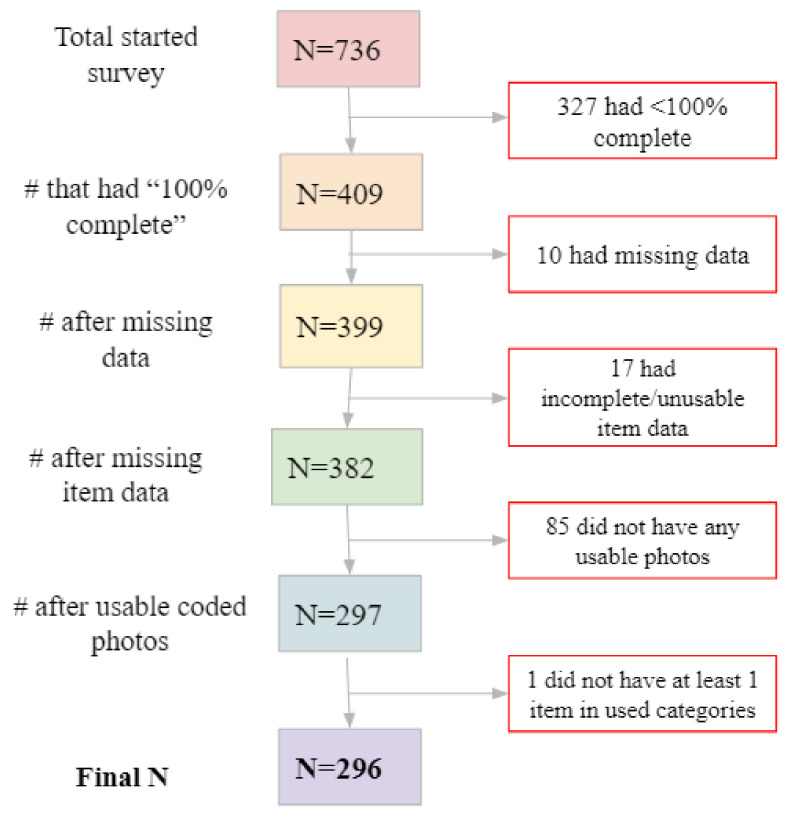
Flow chart with the number of subjects who started the survey and the final number analyzed after excluding missing and incomplete data.

**Figure 3:**
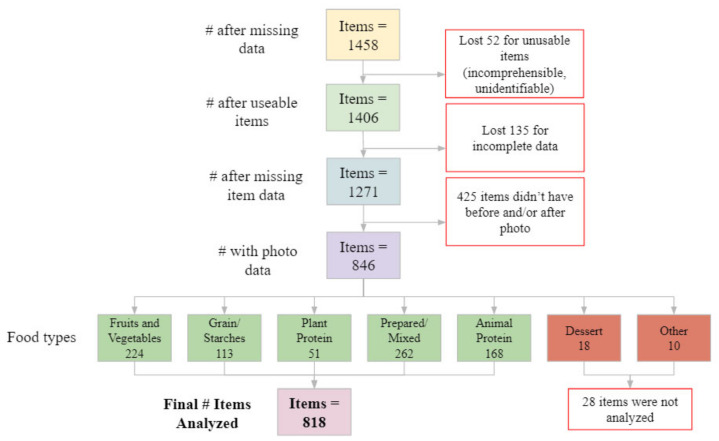
Flow chart with the starting and final number of items analyzed after excluding items with missing and incomplete data.

Responses that were incomplete or incomprehensible were not used. Further, responses relating to food items outside the study’s scope (for example, beverages) were also removed. The food items entered by participants were read and then coded according to seven main categories: Fruits and Vegetables, Grains/Starches, Plant Protein, Prepared/Mixed, Animal Protein, Dessert, and Other. If an item was thought to have two separate components, it was put into the Prepared/Mixed category. Plant Protein was separate from Fruits and Vegetables and included items such as beans, tofu, and plant-based meat. Few items were in the Dessert and Other categories, so they were not included in the analysis. The total number of item responses was 818, which came from a total of 296 subjects. The breakdown of how many subjects and items were discarded at each step is shown in Figure 2 and Figure 3.

For the choice and portion questions that were “check-all-that-apply”, each category was converted into its own response and then each item was coded with a binary value of either zero for “unselected” or one for “selected”. “Pre-plated” and “Someone else served” were combined for a “Pre-plated_combined” category to indicate the instances where a server, rather than the diner, determined the portion quantity.

After their meal, diners were asked if they had finished each item they selected. If the item was not finished, they were asked to select a reason why. This provided an overview of how many items were finished and the self-reported reasons why diners did not finish those other items. To determine the quantity of food wasted, the “before” and “after” photos were examined and coded separately by two staff researchers at Rice University, then checked for agreement. Photos that were either unclear or did not match the survey responses were excluded. The “before” photos were coded for the approximate amount of food in relation to a plate per item mentioned by the diner: 1 tablespoon (1/16 plate), few bites (1/8 plate), 1/4 plate, 1/2 plate, 1 plate. The “after” photos were then coded by the approximate amount of food eaten in relation to the “before” photos, and percentages were assumed for each calculation: fully eaten (100%), mostly eaten (75%), half-eaten (50%), mostly uneaten (25%), and uneaten (0%). Food eaten was approximated by multiplying the percentage eaten in the “after” photo by the amount taken in the “before” photo. Food waste was then approximated in units of “percentage of plate” by subtracting how much was eaten from how much was taken. The amounts of food taken and wasted were standardized into z-scores by school/semester combination to minimize school variation and to normalize the residuals.

### 2.3. Statistical Analysis

RStudio (Version 1.1.463) using R Version 4.0.0 was used to analyze the data. The type I error rate (α) was set at 0.05 when reporting significance.

#### 2.3.1. Multiple Correspondence Analysis (MCA)

For the MCA, the amounts of food taken and wasted were not scaled but rather converted into factors to run the analysis. Personal and situational factors were measured per subject or item to assess correlations with the amount of food taken and wasted. Factors of food type (Type), how confidently a diner thought they would like the item before choosing it (Confidence), frequency of eating at the dining commons (Frequency), satisfaction with meal (Satisfaction), school (School), and disposal category (Disposal) were analyzed with amount taken (Taken), and amount wasted (Wasted) with an MCA. The number of days differed among schools in order to reach the quotas, however, this was not included as a factor because the variation that came from it—different meals and serving styles—was captured in the other factors.

Food waste was measured in two ways: self-reporting categorization and photographic numerical estimation. The MCA was used to assess how these measurements aligned. The “Finished” category was found near the numerical “Waste_0%” factor while reasons for not finishing were located near the numerical “Waste_100%” and “Waste_75%” factors.

#### 2.3.2. Multiple Linear Regression

Multiple linear regressions using the lme4 package were run to investigate the relationship of question responses to the standardized amounts of food taken and wasted, which were treated as continuous factors. Bartlett’s test was used on the standardized food taken and wasted amounts. No significant difference was found in the variance among groups (*p* > 0.05), so the data was combined and analyzed as such. Schools were not analyzed individually since there were unequal numbers of observations per school. Disposal categories were not included in the model since the standardized quantities were of greater interest here. Confidence and satisfaction scores were assumed to be continuous and normal. Food type and frequency of eating at the dining commons were treated as categorical factors. Models that included food type: confidence and food type: satisfaction interactions were also created but were not significantly different from the models without the interaction terms, so they were not used. ANOVA and adjusted R-squared values were used for model comparison.

#### 2.3.3. Analysis of Variance and Post-Hoc Tests

ANOVAs were run to determine if there were significant differences among food types on the amount of food taken and wasted. Fisher’s LSD from the agricolae package was used as a post-hoc test.

#### 2.3.4. Correspondence Analyses

After chi-square tests showed significant differences of distributions in responses among food types and confidence levels, correspondence analyses from the FactoMineR and factoextra packages were run on the check-all-that-apply data. Correspondence analyses were used to relate amount of food taken and food types with the reasons for selecting the portion size. Another correspondence analysis was run to relate confidence of taking items to reasons for selecting the items.

## 3. Results

### 3.1. Factors Affecting the Amount of Food Taken and Wasted

**Figure 4:**
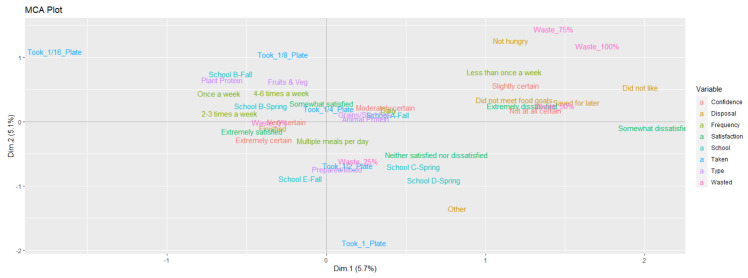
Multiple correspondence analysis (MCA) of personal and situational factors related to the amount of food taken and wasted.

The MCA results (Figure 4) that explain 10.8% of the variation after the first two dimensions, show that not wasting food (“Waste_0%” factor in the MCA) is in the same domain as the factors for going to the dining commons for multiple meals a day, being extremely certain they were going to like the dish before they chose it, and extreme satisfaction with their dish. Associations around wasting more food (“Waste_100%” and “Waste_75%” factors in the MCA) are less closely clustered but include going to the dining commons less than once a week, being less certain they were going to like the dish before they chose it, and extreme dissatisfaction with the dish. Overall, the MCA suggests that having more confidence in choosing the dish before eating it, being satisfied with the dish after eating it, and going to the dining commons more frequently were all positively correlated with the diner finishing the dish.

### 3.2. Factors Affecting the Amount of Food Taken

**Figure 5:**
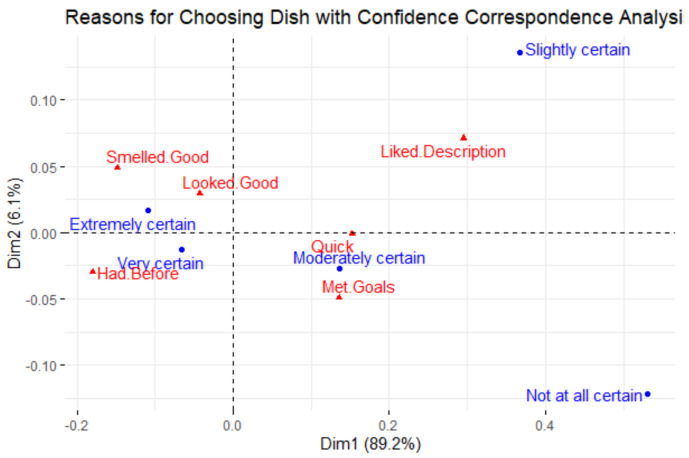
Correspondence analysis relating reasons why participants selected the food and the level of confidence they had in liking it before taking the item.

A linear model was created relating food types, confidence, and satisfaction to the amount of food taken. The coefficients are shown in Table 2. Prepared/mixed items (*p* < 0.01) and animal protein (*p* < 0.05) food types demonstrated a significant positive correlation with the amount of food taken. And perhaps not surprisingly, the diners’ confidence in liking the food before choosing it also had a positive correlation with the amount of food taken (*p* < 0.05). Figure 5 shows the results of the correspondence analysis that relates the reasons why people selected their dish and the confidence they had in liking it before selecting it. After two dimensions, 95.3% of the total inertia (0.025) was explained.

#### Food Type Differences

**Figure 6:**
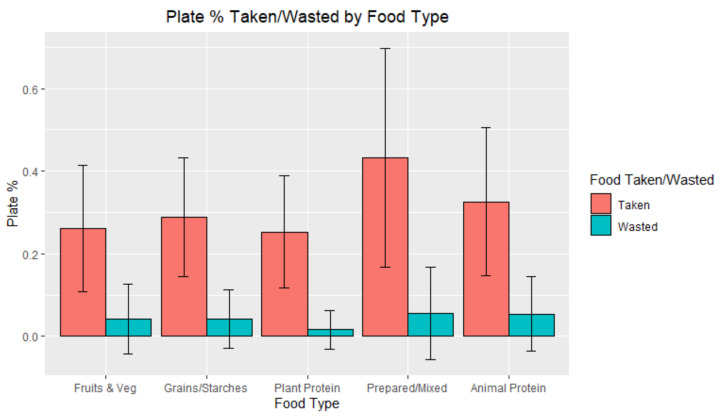
Percent of the plate that was taken and wasted by food type.

The amount of food taken by the diner, measured in percentage of the plate, was significantly different among the food categories (*p* < 0.05), as shown in Table 3 and Figure 6. Prepared/mixed items took up a greater percentage of the plate over animal protein and grains/starches, which took up a greater percentage than fruits and vegetables and plant protein. Meanwhile, the amount of food wasted was not significantly different among the various food categories (*p* = 0.058).

**Figure 7:**
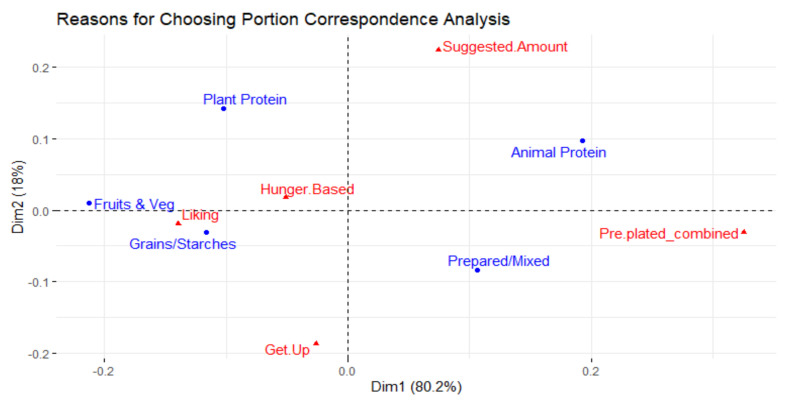
Correspondence analysis relating reasons why participants selected the portion size of the food and the different food types.

Figure 7 shows the results of the correspondence analysis that relates the reasons why people selected the quantity they did to the different food types. After two dimensions, 98.2% of the total inertia (0.032) was explained. Fruits and vegetables and grains and starches are closer together and share the same space of liking the food as the main reason why a particular portion was selected. Animal protein and prepared/mixed dishes are closer to pre-plated and suggested amounts as influencing reasons for selecting the portion size for these food types.

**Figure 8:**
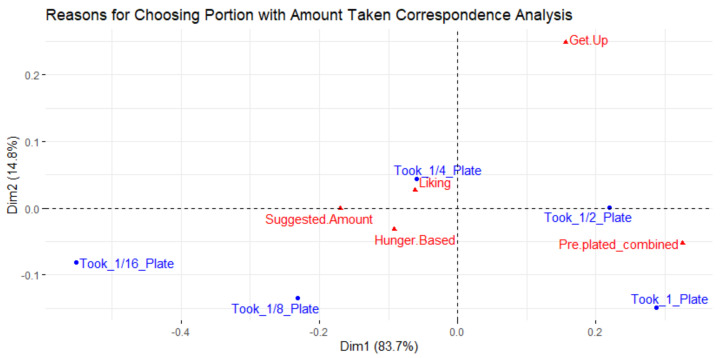
Correspondence analysis relating reasons why participants selected the amount of food and the amount of food taken.

Figure 8 is a correspondence analysis that relates the reasons why people selected the portion they did and the amount of food they took. After two dimensions, 98.2% of the total inertia (0.032) was explained. The greater amounts of food taken (Took_1_Plate and Took_1/2_Plate) are closer to the reason of the item being pre-plated. Food being pre-plated or served by someone else is related to a higher amount of food on a diner’s plate.

### 3.3. Factors Affecting Amount of Food Wasted

A linear model was created to relate standardized amount of food wasted to food type, confidence, satisfaction, and frequency. The coefficients are shown in Table 4. Amount of food taken, satisfaction with the meal, and frequencies of going to the dining commons—2–3 times a week, multiple times a day—were significant predictors of the standardized amount of food wasted (*p* < 0.05). None of the food types were significant predictors which aligned with the previous result that food waste did not significantly differ among food types (*p* > 0.05).

The standardized amount of food wasted was significantly affected by the standardized amount of food taken so there was an indirect impact from factors affecting food taken—food type, confidence. Other direct factors that impacted food waste included satisfaction with the meal and frequency of going to the dining commons. Diners who were more satisfied with their meal wasted less. There was a trend with frequency of going to the dining commons where the more frequent the visit, the less the diner wasted. The 2–3 times a week category was an exception where subjects in that group wasted less on average than subjects in other groups.

## 4. Discussion

### 4.1. Food Waste Drivers

Drivers of food waste have been studied in households but not as much in institutional settings like university dining commons. Drivers in these settings differ since AYCE facilities offer the diner the possibility of getting more food with no financial penalty if it is not finished, which has been linked with higher food waste [35]. Informing households that they could save money by wasting less has been shown to reduce food waste [36], but that does not apply to AYCE dining commons. The significant factors that affected food waste in the model (Table 4) were satisfaction with the dish, frequency of visiting the dining commons, and the amount of food taken.

Higher ratings of satisfaction were related to less food being wasted which aligns with more desired foods—through taste value—being wasted less [37]. However, increasing the appeal of food to reduce food waste is not so straightforward. Consumers have different preferences so changing a dish to fit one person’s taste might reduce satisfaction for others. Offering a wider variety of dishes can be a way to appeal to the diverse group of diners, but this could lead to diners taking more than they need if they exhibit variety-seeking behavior. Diners who visited the dining commons more often tended to waste less, which could be due to them knowing what dishes they like since dining commons often have rotating menus. Data were collected a few weeks into Fall or Spring semesters so it is possible that diners would have already had an idea of which dishes they liked. If data were collected earlier, there might not have been a difference in food waste among different frequency categories. Since the diners and institutions tested differed between Spring and Fall, it was not possible to compare how food waste behavior might have changed over time, an interesting question to investigate in the future. The one institution that was tested in both Spring and Fall collected data at different dining commons for each semester, so their data was also not suitable for an analysis of how behavior changed over time.

A higher amount of food being taken was also related to more food being wasted, most likely due to more waste potential. Animal protein and mixed dishes were found to be taken in significantly higher proportions of the plate (*p* < 0.05). Pre-plated items were in the same space as animal protein and prepared/mixed dishes in Figure 5, and in the same space as taking 100% and 50% of the plate in Figure 7. Due to the lack of controls in this observational study, it is not possible to conclude if food type, method of portioning, or another unidentified reason led to animal protein and mixed dishes taking up a greater portion of diners’ plates on average. Portioning should be considered since less portion control has led to more food waste in previous studies [33,35].

In university dining commons, staple foods, which are considered less valuable, are wasted more [38,39]. In this study, no significant difference was found in the amount of food wasted among the food types (*p* > 0.05). Since most people reported that they finished their food, the lack of variance in plate waste may have prevented the detection of any significant difference. This could be due to underreported food waste values, which can occur when participants are asked to self-report [40]. Food waste behavior also differs in an AYCE setting since financial incentives to finish higher valued products are not present as they are in households [35]. Prompts and signs could be used to remind diners not to waste food, even though there is no financial incentive to do so.

Increased confidence in liking a dish before choosing it also led to a higher amount of it being taken, but less being wasted. In the correspondence analysis relating confidence and reasons for taking items (Figure 5), higher confidence levels were in the same area as having the item before, as well as looking and smelling good. Looking and smelling good are subjective judgments and are difficult to standardize for all diners; however, allowing diners to sample the dish could be a strategy to increase confidence and encourage healthier options. Offering small samples of dishes could help diners decide what they want to choose and thus reduce disappointment after taking a full dish. If this option is pursued, staffing and sample placement must be considered since it can be difficult during busy hours for staff to prepare samples and for diners to obtain these samples [14].

### 4.2. Data Limitations

#### 4.2.1. Sampling

Asking about food waste could cause subjects to feel embarrassed or ashamed if they did waste food and thus might impact their behavior. The study was designed to try to minimize the effects of the survey on food consumption and waste behavior through its pacing, the order of the questions, and by disguising the main purpose of the study; yet there is a possibility that the goal of the study could still have had an effect. There could have also been selection bias where potential participants who tend to waste more might not have volunteered for this food choice study. Personal factors like gender [41], disposable income [39], and education level [39] have also been correlated with food waste in university dining commons but the information was not captured in the survey.

#### 4.2.2. Data Analysis

Running an MCA allowed multiple correlations to be examined at the same time. This type of multivariate analysis is necessary for exploring multifaceted issues such as food behavior. MCA also works well with a range of different data types (for example, categorical, continuous), which aligned well with our data seta. However, the downside of multivariate statistics is that they generally lead to a low percentage of the variance being explained.

This study had a mix of self-reported as well as photographic data which was coded by the researchers. Previous food waste studies have quantified food portions and waste using photographs [33,42] or measured food waste directly [41,43,44]. Having both self-reported and photographic data would have allowed comparisons to see how well the two methods aligned. However, due to most items being finished, it was not possible to carry out a meaningful comparison.

Subjects reported the items and their amounts, so they were not standardized to servings and thus made comparisons difficult. For instance, a participant’s “4 slices of pizza” would have been counted the same as another participant’s entry of “salt.” The condiments/other category was excluded in this analysis for that reason, as well as the lack of items reported in that category.

Photos were used to estimate the proportion of the plate taken up by items. While this allowed the quantification of food taken relative to the plate, there was not enough information to convert items to servings, which might have been be a more meaningful metric. Estimates were also based on photos so there could be variations depending on how items were placed on the plate by the participant. Future studies with options to self-report food items could benefit from asking participants to report servings of items along with specifications of typical serving sizes for popular foods.

In the data organization process, incomplete items had to be removed, which eliminated data on certain items from subjects or all the data from a subject entirely. This reduced the number of observations from 1406 to 818, as seen in Figure 3. The greater number of items would have strengthened the analyses and could have helped increase model fit. Since different numbers of items were deleted for different diners, conclusions could not be drawn regarding how many items each person selected.

Linear models were created to explore a range of factors that might explain the amount of food that was taken and wasted. When it comes to decision making, there are a multitude of factors to consider, each with much variation, so modeling human behavior usually results in low model fit [33,45]. There was an especially low R-squared value for the amount of food wasted model. This could be due to the skewed distribution observed in the amount of food wasted, likely a result of most subjects finishing their food. Standardizing the data was an attempt to correct this skew by normalizing the residuals. Since food waste amounts were converted to z-scores per school, school to school variation could not be studied. This was acceptable for this study since the data was analyzed together, and the individual school was not used as a factor aside from preliminary analysis in the MCA.

Due to the data structure, the check-all-that-apply responses for why items and their portion sizes were chosen were analyzed using correspondence analyses which can only compare two variables at a time. The check-all-that-apply responses were related to food type and the amount of food taken. These analyses elucidated why certain food types were taken as well as why they were taken in larger amounts. However, with these analyses, it is not possible to differentiate the effects of the variables. For instance, it is not possible to know if a greater amount of food being taken was due to it being a mixed/prepared dish or pre-plated dish since the analysis only enables two variables to be examined at a time.

This study included multiple campuses and various times of data collection, which helps with generalization—which has been an issue raised in previous studies [46]. Though this increased the sample size and relevance to multiple schools, this also increased the amount of variation due to factors such as student population and menus. All the participating schools had an AYCE system, which allowed for some comparisons. The same researchers coded all the photos to also reduce noise. The plate amounts were standardized to z-scores by school and those values were used as the dependent variable to further reduce the variation due to differences in school/dining hall/etc. Future experiments that investigate the drivers of food choice and waste or that conduct interventions to reduce food waste should consider multi-campus studies to ensure findings can be generalized outside that institution. There also needs to be a way to properly deal with the variation that could appear as noise when analyzing the data. This could be reduced by collecting additional situational information (participant interest in the menu, number of people they were with, how busy was the dining commons) and of the dining environment (proximity of seats to dining stations, method of dish return), which has also been found to affect behavior [14,37].

## 5. Conclusions

University all-you-care-to-eat dining commons are a well-suited environment to study and understand free-choice food choice and waste behavior. For food choice, prepared/mixed and animal protein items took up a greater percentage of the plate compared to other food types. These items were correlated to the more hedonic-driven reasons for selection and to pre-plated servings. Healthier items such as fruit and vegetables and plant protein dishes took up a smaller percentage of the plate, comparatively. These items were correlated to the reasons of meeting goals and being self-served.

Food waste did not significantly differ among the food types but was related to the amount of food diners took, how satisfied they were with their meal, and how often they went to the dining commons. An increase in the amount of food taken was correlated with an increase in waste. The more satisfied diners were with their meal, the less they wasted. Increased frequency in visiting the dining commons tended to decrease the amount of food wasted.

Future studies could investigate other recruitment methods that reduce selection bias for a more representative sample of food waste behaviors. Researchers should also be mindful of survey design to prevent missing data whenever possible. Questions should be designed with data analysis methods in mind to avoid having to convert continuous data to categories. Building questions off key factors from existing behavior literature could help capture more variation and increase model fit. Different modeling techniques such as using Bayesian, generalized linear models, or structural equation modeling could be used to deal with non-normal data, set subjects as random variables, and examine multiple variables’ relationships with each other and to latent variables, respectively.

This multi-campus study provided insight into drivers of university dining common food choice and food waste as well as ideas for the conduct of future studies. By understanding drivers, universities can work with their dining commons and students to get diners to eat healthier and waste less.

## Figures and Tables

**Table 1:** Characteristics of the schools which participated in this study.

Characteristic	School A	School B	School C	School D	School E
Undergraduate population (%)	90	45	76	90	60
Female (%)	54.3	54.7	51.7	57.9	55.7
Private/Public	Private	Private	Public	Public	Public
Population density	Suburban	Suburban	Urban	Suburban	Suburban
Quarter/Semester observed	Fall	Spring, Fall	Spring	Spring	Fall
Plate size (in.)	10	9.5	9.5 × 6.75	9	9
All you care to eat	Yes	Yes	50%	25%	Yes
Operator served—plated	25–30%	Action stations	50%	75%	Most are operator served

**Table 2:** Model predicting standardized amount of food taken—regression results.

	Estimate	Std. Error	*t* Value	Pr (>|t|)
(Intercept)	−0.71427	0.18284	−3.906	0.000101
Grains/Starches	0.09782	0.10999	0.889	0.374060
Plant Protein	−0.03517	0.14730	−0.239	0.811329
Prepared/Mixed	0.70146	0.08641	8.118	1.75 × 10^−15^
Animal Protein	0.28342	0.09686	2.926	0.003527
Confidence	0.09183	0.03415	2.689	0.007314
Satisfaction	0.01543	0.03954	0.390	0.696356

Residual standard error: 0.9475 on 811 degrees of freedom. Multiple R-squared: 0.1034, adjusted R-squared: 0.09675. F-statistic: 15.58 on 6 and 811 DF, *p*-value: <2.2 × 10^−16^.

**Table 3:** Means and standard deviations were calculated for each food category for the amount taken and wasted in the percentage of the plate. The number of responses for each category is also listed. Different letter means sharing superscript letters were not significantly different as per Fisher’s LSD (*p* < 0.05).

Food Category	*n*	Amount Taken (% Plate)			Amount Wasted (% Plate)	
		Mean		Std Dev	Mean	Std Dev
Fruits and Vegetables	224	0.260	c	0.153	0.041	0.085
Grains/Starches	113	0.288	bc	0.144	0.041	0.071
Plant Protein	51	0.252	c	0.136	0.016	0.047
Animal Protein	168	0.326	b	0.178	0.054	0.113
Prepared/Mixed	262	0.432	a	0.266	0.055	0.090

**Table 4:** Model predicting standardized amount of food wasted regression results.

	Estimate	Std. Error	*t* Value	Pr (>|t|)
(Intercept)	1.14197	0.32328	3.532	0.000435
Std_Taken	0.32810	0.03491	9.399	<2 × 10^−16^
Grains/Starches	−0.10930	0.10976	−0.996	0.319620
Plant Protein	−0.26305	0.14613	−1.800	0.072221
Prepared/Mixed	−0.16809	0.08937	−1.881	0.060362
Animal Protein	0.01615	0.09671	0.167	0.867459
Confidence	−0.02684	0.03418	−0.785	0.432509
Satisfaction	−0.10252	0.03945	−2.599	0.009532
Once a week	−0.38276	0.30338	−1.262	0.207438
2–3 times a week	−0.60250	0.29668	−2.031	0.042601
4–6 times a week	−0.46817	0.28856	−1.622	0.105103
Daily	−0.49057	0.28057	−1.748	0.080762.
Multiple meals a day	−0.58855	0.27661	−2.128	0.033664

Residual standard error: 0.9393 on 805 degrees of freedom. Multiple R-squared: 0.1252, adjusted R-squared: 0.1122. F-statistic: 9.605 on 12 and 805 DF, *p*-value: <2.2 × 10^−^^16^.

## Supplementary Materials

The following are available online at https://www.mdpi.com/2304-8158/10/3/577/s1, Table S1. The questions that were asked in the order they were presented in the survey.
