# Supplementary material for: Food Choice and Waste in University Dining Commons—A Menus of Change University Research Collaborative Study
Source: Foods. 2021 Mar 10;10(3):577. doi: 10.3390/foods10030577 (PMC8000212; doi:10.3390/foods10030577)
Supplement: Supplementary file 1 [file foods-10-00577-s001.pdf]

Table S1. The questions that were asked in the order they were presented in the survey.

Info	Question	Asked Per	Question Type	Options
Before Photo	Please take a picture of your plate (before you start eating), and upload it using the button below. If you have more than one plate, please try to take a picture of all of your food together. Don't worry about taking a picture if you go back for more, we will ask you about that in the questions that follow.	Person	Photo	N/A
Items	Please tell us what you chose to eat today (in your words, so we can use it for subsequent questions). If you have more items than spaces for them, please just write the most significant ones in the spaces provided. Please focus on main dishes and sides (rather than drinks or desserts). Please write down at least one item, and up to six different items.	Person	Open-Ended	N/A
Reason	Please tell us why you chose the items on your plate today. Select all that apply:	Item	Check-all-that-apply categorical	Looked good<br>Smelled good<br>Liked Description<br>I've had before<br>It was quick<br>Met my nutritional goals<br>Other
Choice	Please tell us how you chose the quantities of food you took today. Select all that apply:	Item	Check-all-that-apply categorical	Pre-plated<br>Someone else served<br>Suggested amount<br>Based on my hunger<br>Liking of food<br>I didn't want to get up again<br>Other
Confidence	How confident were you that you would like this item before you took it?	Item	Ordered categorical	Not at all certain<br>Slightly certain<br>Moderately certain<br>Very certain<br>Extremely certain
Go Back	How likely are you to go back for more (of any item) if you like it?	Person	Ordered categorical	Extremely unlikely<br>Somewhat unlikely<br>Niether likely nor unlikely<br>Somewhat likely<br>Extremely likely
After Photo	Please take a picture of your plate when you are finished eating, and upload it using the button below.	Person	Photo	N/A

	Please try to show us whatever food is left on the plate.			
Disposal	Why did you choose not to finish (if you did not) each of the following items?	Item	Categorical	Did not like<br>Not hungry<br>Did not meet food goals<br>Saved for later<br>Other<br>Finished
Satisfaction	How satisfied were you with your meal today?	Person	Ordered categorical	Extremely dissatisfied<br>Somewhat dissatisfied<br>Neither satisfied nor dissatisfied<br>Somewhat satisfied<br>Extremely satisfied
Role	Are you:	Person	Categorical	Student<br>Staff<br>Faculty<br>Visitor<br>Other
Frequency	How often do you eat in the dining hall?	Person	Ordered categorical	Less than once a week<br>Once a week<br>2–3 times a week<br>4–6 times a week<br>Daily<br>Multiple meals per day
